# Organoids as next-generation models for investigating intracranial tumours

**DOI:** 10.1186/s13041-026-01317-y

**Published:** 2026-05-30

**Authors:** Subham Roy, Fahmida Zahin, Princess Afia Nkrumah-Boateng, Saim Chaudhry, Maher Nassor, Maame Fosuah Appiah Kwarteng, Akosua Bempomaa Owusu-Boampong, Andrew Awuah Wireko

**Affiliations:** 1 Hull York Medical School, University of York, York, UK; 2 The University of Buckingham Medical School, Buckingham, UK; 3 University of Ghana Medical School, Accra, Ghana; 4 The University of Edinburgh Medical School, Edinburgh, UK; 5 School of Medicine, University of Sunderland, Sunderland, England; 6 Department of Biochemistry and Molecular Biology, Wright State University, Dayton, USA; 7 Kumasi Centre for Collaborative Research in Tropical Medicine, Kumasi, Ghana; 8 Department of Research, Toufik’s World Medical Association, Sumy, Ukraine

**Keywords:** Brain organoids, Brain tumour organoids, Intracranial tumours, Disease modelling, Precision neuro-oncology

## Abstract

Tumour organoids have emerged as important tools in brain tumour research, addressing long-standing limitations of conventional two-dimensional cultures, xenograft models, and genetically engineered mouse models. By preserving patient-specific genetic alterations, cellular diversity, spatial architecture, and key microenvironmental features, organoid systems enable more faithful modelling of tumour biology across a broad range of intracranial tumours, including gliomas, meningiomas, medulloblastomas, pituitary tumours, and craniopharyngiomas. Patient-derived organoids, genetically engineered models, co-culture systems, and bioprinted platforms have collectively advanced understanding of tumour initiation, invasion, heterogeneity, and therapeutic resistance, while offering clinically relevant systems for drug screening and personalised therapy prediction. Importantly, organoid models facilitate mechanistic interrogation of tumour–microenvironment interactions that are difficult to capture in other systems, including neural–tumour crosstalk, vascular niche formation, and immune modulation. Their compatibility with high-throughput screening and integration with emerging technologies, such as single-cell and spatial omics, CRISPR-based genome editing, microfluidics, and artificial intelligence, has further expanded their utility for functional genomics, biomarker discovery, and predictive modelling of treatment response. The development of large-scale organoid biobanks that represent diverse tumour subtypes and patient populations also provides critical infrastructure for reproducible research and collaborative precision oncology efforts. While challenges remain, including variability in culture protocols, incomplete immune and vascular representation, and barriers related to cost and technical complexity, ongoing methodological innovations are progressively enhancing the physiological fidelity and translational relevance of organoid systems. Overall, tumour organoids represent a promising interface between experimental research and clinical application in neuro-oncology, with significant potential to accelerate therapeutic discovery, refine patient stratification, and ultimately improve outcomes for individuals with brain tumours.

## Introduction

Intracranial tumours are neoplasms that develop within the cranial cavity, originating from structures such as the brain parenchyma, meninges, cranial nerves, the sellar region, and other intracranial components. These tumours represent a diverse group, including gliomas, embryonal tumours like medulloblastoma, meningiomas, pituitary tumours, nerve sheath tumours, and craniopharyngiomas. [1] They are classified based on their histological characteristics and, increasingly, their molecular features. They represent a major source of cancer-related morbidity and mortality across all age groups [2]. A recent global burden of disease analysis indicates that all central nervous system (CNS) tumours account for a disproportionate burden of disability-adjusted life years relative to their incidence, reflecting high mortality rates, long-term neurological sequelae, and limited therapeutic options, particularly for high-grade malignancies such as glioblastoma [3].

Despite advances in surgical techniques, radiotherapy, and systemic therapies, the prognosis for many intracranial tumours remains poor, with glioblastoma exhibiting a median survival of approximately 15 months and near-universal recurrence [4]. A major obstacle in improving outcomes lies in the biological complexity of brain tumours, which display profound intratumoural heterogeneity, dynamic clonal evolution, therapy resistance, and intricate interactions with the neural and immune microenvironment [5, 6]. These features are difficult to capture using conventional experimental models, contributing to the high attrition rate of neuro-oncology therapeutics during clinical translation.

Traditional two-dimensional (2D) cell culture systems have historically served as foundational tools for studying tumour biology and screening anticancer agents. However, prolonged in vitro passaging leads to genetic drift, loss of tumour-specific transcriptional programmes, and failure to preserve spatial organisation and cellular diversity, thereby limiting their translational relevance [7, 8]. For example, animal models, including xenografts and genetically engineered mouse models (GEMMs), partially overcome these limitations by enabling in vivo tumour growth and therapeutic evaluation. Nonetheless, xenograft models are confounded by species-specific differences in brain architecture, immune responses, and extracellular matrix (ECM) composition, while GEMMs are restricted to predefined oncogenic drivers and fail to capture the breadth of patient-specific heterogeneity observed clinically [3, 9]. Collectively, these shortcomings underscore the need for human-relevant, scalable, and biologically faithful models to study intracranial tumours and accelerate translational discovery.

Brain organoids are three-dimensional (3D) in vitro structures generated from pluripotent stem cells (PSCs) that self-organise into region-specific neural tissues, closely mimicking key aspects of human brain development, cytoarchitecture, and cellular diversity [10]. Building upon this platform, brain tumour organoids (BTOs) are derived either directly from patient tumour specimens or through genetic manipulation of neural organoids to introduce tumour-associated alterations, enabling the modelling of oncogenesis within a human neural context [8, 11]. Unlike conventional cultures, tumour organoids preserve tumour-specific genetic alterations, epigenetic states, and transcriptional programmes, while maintaining heterogeneous populations of cancer stem-like cells, progenitors, and differentiated tumour cells [5, 7]. Importantly, they retain aspects of the tumour microenvironment (TME), including ECM components, hypoxic gradients, and, in some systems, immune and endothelial interactions [12, 13].

Tumour organoids represent a paradigm shift in neuro-oncology research by offering a platform that more faithfully mimics human tumour biology than existing in vitro or in vivo models [6]. Their ability to preserve patient-specific molecular features enables functional interrogation of tumour heterogeneity, clonal evolution, and mechanisms of therapy resistance that are otherwise obscured in reductionist systems [4]. From a translational perspective, organoids facilitate medium- to high-throughput drug screening, allowing rapid assessment of therapeutic vulnerabilities and resistance patterns in a patient-specific manner [14]. Furthermore, recent advances have demonstrated their utility in evaluating immunotherapies, including chimeric antigen receptor T (CAR‑T) cells and immune-modulating agents, as well as in integrating multi-omics and spatial profiling technologies to dissect tumour ecosystems at unprecedented resolution [6, 15].

The aim of this review is to critically evaluate the strengths and limitations of brain tumour organoids, discuss technical and conceptual challenges that remain, and outline future directions for their integration into translational neuro-oncology research and clinical decision-making.

## Methodology

This narrative review was conducted using the Scale for the Assessment of Narrative Reviews (SANRA) [16] to critically evaluate current and emerging applications of organoid-based technologies in intracranial tumour research. The review focused on methodological advances, biological insights, and translational applications of organoids in intracranial tumours. The detailed search string provided in the appendix enabled a comprehensive and targeted review of the literature to be conducted.

### Eligibility criteria

The inclusion criteria focused on experimental and translational studies involving “patient-derived organoids”. Clinical studies were usually employed when organoids were used for drug testing, disease modelling, and personalised medicine. The review included articles published in English only, with the timeframe for selecting these papers being from inception until 2026. Conference abstracts without full papers, non-peer-reviewed studies, preprints, editorials, letters and blog posts were excluded from the review.

### Search strategy

The literature search was conducted using databases such as PubMed/Medline, Scopus, Embase and the Cochrane Library. Medical Subject Headings (MeSH) and Boolean operators (AND and OR) were utilised when searching the databases, as shown in the following: ("Organoids"[Mesh] OR organoid* OR "tumour organoid*" OR "patient-derived organoid*" OR "cerebral organoid*" OR "brain organoid*" OR "3D culture*" OR "three dimensional culture*" OR "three-dimensional culture*") AND ("Brain Neoplasms"[Mesh] OR "Central Nervous System Neoplasms"[Mesh] OR glioma* OR glioblastoma* OR medulloblastoma* OR meningioma* OR craniopharyngioma* OR "pituitary tumour*" OR "pituitary neoplasm*" OR pituitary adenoma* OR "intracranial tumour*" OR "brain tumour*") AND (model* OR "disease model*" OR "tumour microenvironment" OR "drug screening" OR "drug testing" OR "drug response" OR "precision medicine" OR "targeted therapy"). This approach ensured that the literature search targeted our specific area of interest.

### Article selection and assessment

**Fig. 1:**
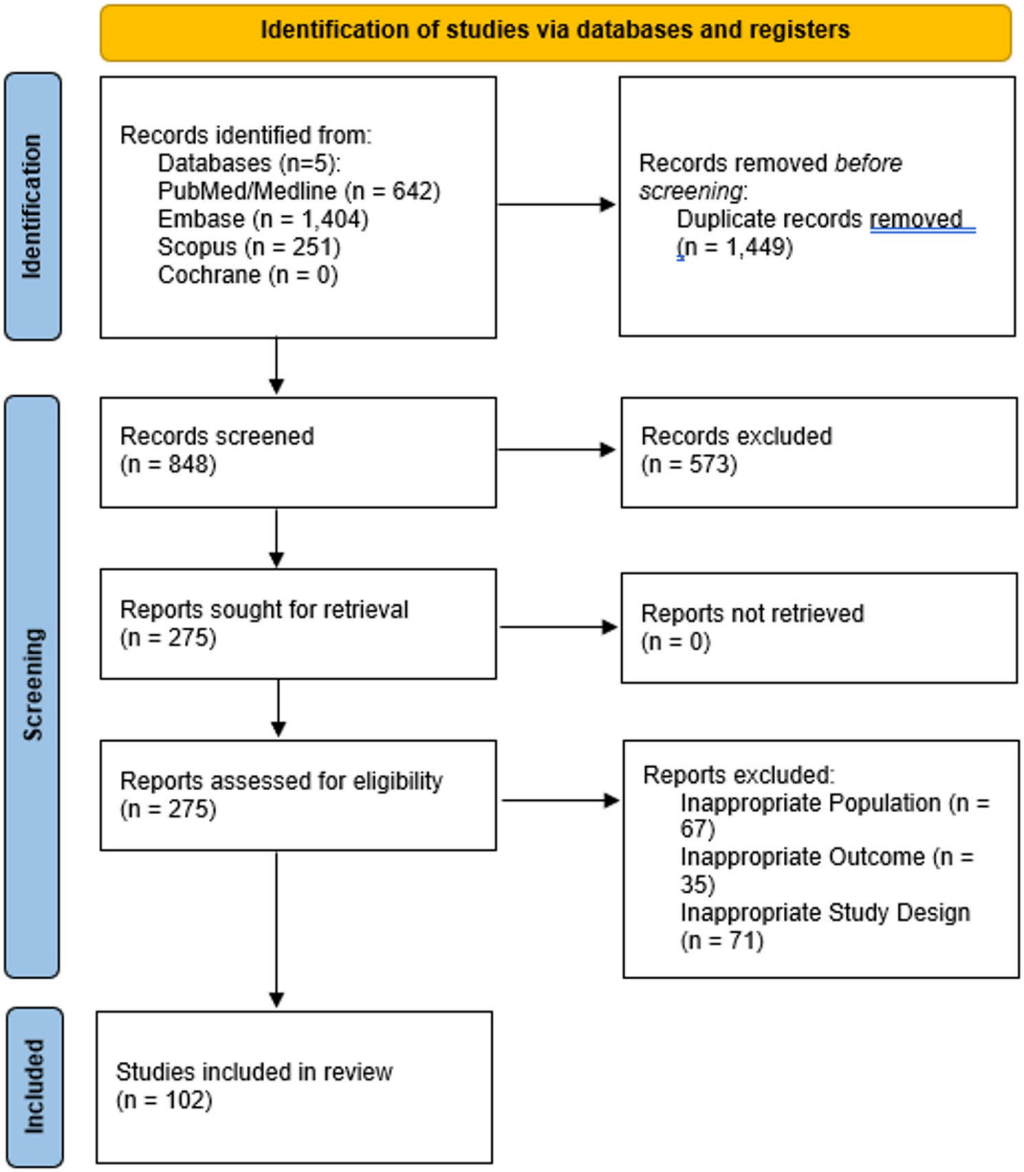
A flow chart for the article selection process. A literature search was conducted using the databases Pubmed/Medline, Embase, Scopus and the Cochrane Library. 2,297 articles were obtained from the databases, of which 1,449 duplicates were removed. 848 articles underwent screening using the inclusion and exclusion criteria. A total of 102 eligible articles were included in the main results of the review after exclusions during title and abstract screening (n = 573) and full text screening (n = 173)

Duplicated articles obtained from the databases were removed using Endnote 2025. Four authors (S.R., P.A.N.B, S.C., A.A.W) independently reviewed the articles during title and abstract screening and full-text screening, in accordance with the inclusion and exclusion criteria. The senior author (A.A.W.) discussed with the reviewing authors and made the final decision in instances of disagreements regarding the inclusion of articles in this review. Additionally, to improve comprehensiveness, a manual search was conducted to identify references listed in recently published case-specific reviews, providing further insight into the field of organoids and intracranial tumours. Of the 2,297 articles obtained from the databases, 1,449 duplicates were removed and 848 articles were reviewed by the authors. A total of 102 eligible articles were included in the main results of this review for a narrative synthesis. A summary of the methodology is provided in Fig. 1.

## Key approaches to brain organoids and BTO development

### Patient-derived models

Patient-derived cerebral tumour organoids serve as a valuable platform for modelling brain tumours due to their ability to preserve vital histopathological and molecular properties of the original tumour and to more accurately represent the characteristics of human tumours. As a result, these models may improve the predictive relevance of drug efficacy testing in preclinical settings [17]. Patient-derived organoids (PDOs) are developed by culturing tumour cells obtained from several sources, including patient biopsies, surgical specimens, and biological fluids such as blood or ascites [18]. Following tissue acquisition, the tumour material is often subjected to mechanical dissociation, enzymatic dissociation, or a combination of both, resulting in a suspension of isolated cells. These cells are subsequently embedded within ECM domes and cultured in specifically enriched media to support three-dimensional growth and long-term viability. An alternative developmental strategy involves introducing tumour heterozygous alterations via genetic engineering in various substrates, including PSCs, induced pluripotent stem cells (iPSCs), embryonic stem cells (ESCs), normal organoids, or tissue-specific stem cells [18, 19].

As a consequence of these developmentally informed approaches, PDOs can be applied to drug screening, immunotherapy testing, biobanking, and the investigation of brain tumour mechanisms, including cancer stem cells and therapy resistance. These organoids serve as a humanised platform for assessing treatment effectiveness and potential side effects in a biologically relevant context [3]. Importantly, they reflect both the genotype and phenotype of the original tissue while preserving cellular heterogeneity and structural architecture, features that are essential for reliable research and the advancement of personalised therapeutic strategies [20]

### Genetic engineering

3D BTOs can be developed by genetically engineering non-malignant brain organoids to induce cancer-causing mutations and cell proliferation, most commonly using CRISPR-Cas9 technology. This technology is used to engineer specific gene alterations in organoids by designing single-guide ribonucleic acids (sgRNAs) that target specific genes, leading to base-pair insertions or deletions and the induction of mutations in target genes [21]. Through the introduction of these genetic alterations during organoid development, non-malignant brain organoids undergo neoplastic transformation while maintaining aspects of 3D brain architecture.

CRISPR-Cas9 technology has been used to model brain tumours, including glioblastomas and medulloblastomas, by driving tumour formation within genetically engineered cerebral organoids. Neoplastic cerebral organoids (neoCORs) and engineered glioblastoma organoids (eGBOs) replicate brain tumour formation through CRISPR-Cas9-mediated genetic mutations and transposon-mediated gene insertion, resulting in sustained cell proliferation and invasive growth patterns in a 3D context [22–24]. These genetically engineered organoids demonstrate how sufficiently defined oncogenic mutations are to initiate and support tumour development within brain-like tissue.

Specific oncogenic drivers, such as c-MYC and OTX2, were shown to induce group 3 medulloblastoma-like formation and proliferation when introduced into non-malignant human cerebellar organoids, highlighting the role of targeted genetic alterations in tumour specification [22, 25]. Similarly, CRISPR-Cas9 targeting of an HRASG12V-IRES-tdTomato construct was used to develop a 3D model of gliomas in human cerebral organoids, inducing neoplastic transformation characterised by invasive properties and disruption of surrounding organoid structures [26]. Together, these approaches illustrate how genetic engineering enables the developmental generation of brain tumour organoids that mirror key features of tumour initiation and progression.

### Co-culture systems and assembloids

Co-culture systems have been developed to facilitate the investigation of complex interactions between distinct cell populations and to enable a deeper understanding of disease pathogenesis. These systems are particularly valuable for exploring cell–cell and cell–microenvironment crosstalk, thereby enabling researchers to more closely simulate in vivo conditions and study biologically relevant interactions that are not adequately captured by conventional in vitro models. Co-culture approaches have also been widely applied in studies of drug response, therapeutic target identification, and disease modelling [27]. In addition, they have been used to investigate cell differentiation, cellular function and viability, proliferation and migration, CNS development, and metabolic mechanisms [27]. Compared with traditional 2D culture systems, 3D co-culture models provide a more physiologically relevant platform.

The establishment of brain tumour co-culture systems typically involves generating cerebral organoids from PSCs, such as human induced pluripotent stem cells (hiPSCs) or human embryonic stem cells (hESCs), followed by their integration with tumour spheroids, patient-derived glioma stem cells (GSCs), or tumour organoids. Tumour cells are often labelled using reporters such as green fluorescent protein (GFP) or luciferase to enable real-time tracking of tumour invasion into the surrounding neural tissue. These cultures are able to more accurately replicate the in vivo brain cell-microenvironment interactions [28].

Biologically, co-culture systems have proven particularly useful in modelling tumour invasion, as tumour cells infiltrate the organoid matrix in a manner that closely resembles invasion patterns observed in the human brain [29]. They also provide insight into tumour–host interactions, including dynamic communication between tumour cells and neural elements, which is critical for understanding tumour progression and therapeutic responses [30]. Furthermore, these systems support the maintenance of tumour heterogeneity, as patient-derived tumour cells preserve genetic and phenotypic features more effectively than those cultured in 2D systems [31]. In contrast, conventional 2D cultures and simple tumour spheroids often exhibit relatively homogeneous cell populations and lack the complex cell–cell and cell–matrix interactions necessary for sustaining tumour heterogeneity and adaptive plasticity.

By allowing glioblastoma cells, particularly patient-derived glioblastoma stem-like cells, to interact dynamically with a host neural environment, cerebral organoid co-culture models more closely mimic human brain architecture and developmental cues [28, 31]. A notable example is the cerebral organoid glioma (GLICO) model, in which GFP-labelled patient-derived GSCs are integrated into mature cerebral organoids. This approach preserves key genetic and signalling characteristics of the original patient tumours that are frequently lost in traditional 2D cultures. These findings suggest that the complex 3D microenvironment provided by organoids supports phenotypic diversity and cell-state plasticity, both of which are fundamental features of tumour heterogeneity, through sustained interactions with brain-like cellular contexts [31].

Assembloids represent a specialised system in which multiple organoid or cell-type–specific structures are combined to recreate interactions between distinct tissues or brain regions. These models enable the study of complex multicellular dynamics and inter-regional communication that more closely resemble physiological conditions [32]. By integrating different cell populations within a 3D architecture, assembloids allow researchers to investigate biologically relevant processes involved in development and disease pathogenesis while overcoming some of the limitations associated with conventional in vitro models [32].

### Bioprinting

3D bioprinting is an advanced biofabrication approach in which a “bioink” is deposited layer by layer to generate precisely organised 3D tissue constructs that mimic the architecture and functional characteristics of native tumours. Bioinks typically consist of living cells combined with ECM components, enabling the formation of biologically relevant tumour models [33]. Bioprinting technologies commonly employ extrusion-based, inkjet, or laser-assisted mechanisms to deposit cell-laden bioinks into predefined geometries. Tumour cells are often embedded within ECM-like hydrogels and combined with supporting cell populations, such as vascular or stromal cells, to produce structurally complex and reproducible tumour organoid models [33].

Recent advances in bioink engineering have led to the development of materials that offer rapid gelation, mechanical stability, and compatibility with high-throughput platforms. These innovations allow bioprinted tumour organoids to maintain high viability while remaining suitable for downstream applications such as drug evaluation [34]. Furthermore, 3D bioprinting enables the standardisation of tumour models, facilitating automated, scalable drug-screening workflows that are difficult to achieve with manually generated organoid cultures [35]. By integrating patient-derived tumour cells with engineered hydrogels, bioprinted platforms can retain the genetic and phenotypic features of individual tumours, thereby supporting more precise drug-sensitivity profiling and personalised therapeutic approaches [35].

**Fig. 2:**
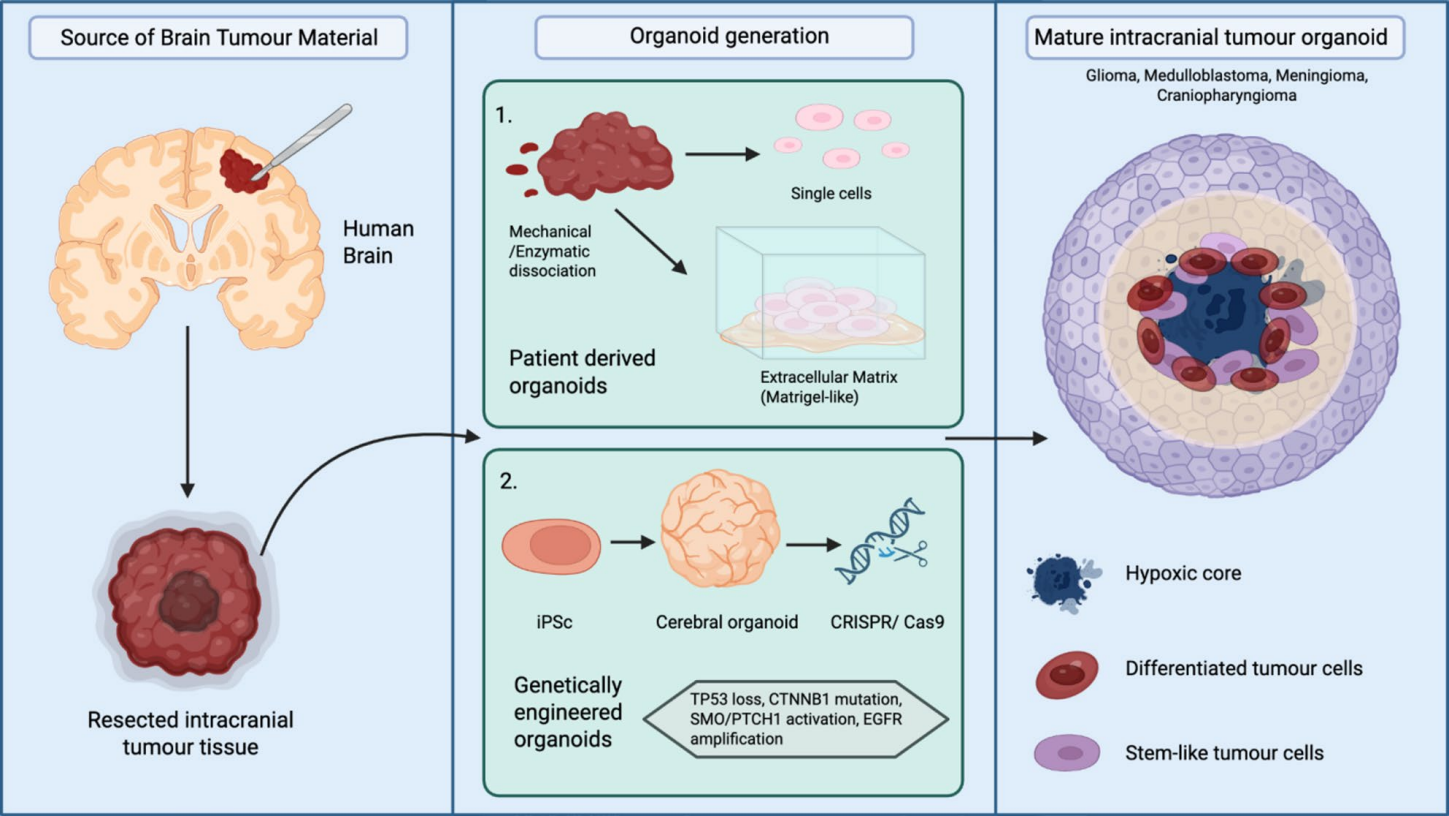
Brain Tumour Organoid Development and Sources (Image is created with Biorender.com). Tumour tissues obtained from surgical resections can be dissociated to obtain patient-derived tumour organoids embedded in an extracellular matrix scaffold. Alternatively, induced pluripotent stem cell (iPSC)-derived cerebral organoids can be genetically engineered to introduce tumour-associated genetic mutations using a CRISPR/Cas9 system. The above methodologies yield mature tumour organoids that capture essential aspects of brain tumours. The methodologies are being used to model diverse intracranial tumours such as gliomas, medulloblastomas, meningiomas, craniopharyngiomas, among others. iSPC, Induced Pluripotent Stem Cell; CRISPR, Clustered Regularly Interspaced Short Palindromic Repeats

In addition to scalability and reproducibility, bioprinting allows for precise spatial patterning of cells and matrix components, enabling the generation of tumour organoids with defined shapes, sizes, and internal organisation, thereby improving experimental consistency [33]. Hybrid strategies that combine bioprinting with organoid technology further support the establishment of controlled microenvironments that reflect physiological conditions, including nutrient and oxygen gradients that are essential for tumour growth and drug response studies [36]. These approaches are particularly advantageous for large-scale drug screening applications, where the ability to generate uniform tumour organoids in high numbers is critical [36]. Figure 2 depicts BTO development and sources.

## Applications of organoid technology in brain tumour research

### Gliomas

#### Establishment and genomic fidelity of glioma organoid models

Patient-derived glioma organoids provide a 3D platform that preserves the genomic architecture, epigenetic states, and clonal complexity of gliomas far more effectively than 2D cultures. Early foundational work demonstrated that glioblastoma organoids generated from minimally processed surgical specimens retain key driver alterations, including *EGFR* amplification and *EGFRvIII* rearrangements, *PTEN* loss, *TP53* mutations, homozygous *CDKN2A/B* deletion, and chromosome 7 gain/10 loss, with strong concordance to matched patient tumours across whole-exome sequencing and copy-number profiling [37]. Furthermore, these organoids preserved both dominant and minor subclonal populations, enabling faithful modelling of intratumoural evolutionary dynamics rather than selection of culture-adapted clones [37, 38].

Lower-grade glioma (LGG) organoids have similarly been shown to retain hallmark genetic and epigenetic features, including *IDH1/IDH2* mutations, *ATRX* loss, and 1p/19q co-deletions, as well as the associated CpG island methylator phenotype that is typically lost in monolayer cultures [39, 40]. Additionally, organoid models derived from stereotactic biopsy specimens have demonstrated the feasibility of preserving clinically relevant alterations, such as TP53, ATRX, and PDGFRA mutations, even when tissue availability is limited, highlighting their translational applicability in real-world clinical settings [41]. Transcriptomic analyses across multiple platforms confirm long-term stability of core oncogenic programmes driven by *SOX2*, *OLIG2*, *MYC*, and *STAT3*, supporting reproducibility and scalability for downstream functional studies [42, 43].

Despite these promising findings, most genomic fidelity studies have been conducted in relatively small cohorts and within specialised research laboratories, which may limit reproducibility across centres and tumour subtypes. Variability in organoid derivation methods, including differences in extracellular matrix composition, culture medium formulations, oxygen gradients, and passaging strategies, can influence tumour cell selection and transcriptomic stability [4, 20]. This methodological heterogeneity complicates direct comparison across studies and highlights the need for protocol standardisation and multi-centre validation. Furthermore, large-scale benchmarking studies evaluating genomic concordance between parental tumours and derived organoids across diverse patient populations remain limited. Consequently, although glioma organoids demonstrate strong preservation of tumour genomic architecture, including driver mutations and clonal diversity, further validation in larger prospective cohorts is required before these models can be reliably implemented in clinical precision oncology workflows [3, 5, 8]

Recent advances in genetically engineered brain organoids have enabled direct modelling of glioblastoma initiation and progression through targeted introduction of oncogenic alterations, including *TP53* loss, *PTEN* deletion, *EGFRvIII* expression, and *CDKN2A* loss, within defined developmental contexts [24]. These engineered systems recapitulate spatial and temporal transitions from proliferative progenitor-like states to invasive mesenchymal programmes, providing causal links between genotype, developmental timing, and tumour architecture [24]. Collectively, patient-derived and genetically engineered organoids establish a continuum of models spanning tumour initiation, progression, and heterogeneity.

However, genetically engineered organoid systems often rely on predefined oncogenic alterations introduced via CRISPR-Cas9 or other genome-engineering strategies. While these models provide valuable mechanistic insight into tumour initiation and developmental timing, they may oversimplify the highly complex mutational landscapes and evolutionary trajectories observed in spontaneous human gliomas. In clinical tumours, glioblastoma evolution is shaped by cumulative genomic instability, TME interactions, and therapy-induced selective pressures, which are difficult to fully reproduce in engineered organoid systems. As a result, findings derived from genetically engineered models must be interpreted cautiously when extrapolating to heterogeneous patient populations [6, 8, 22, 24].

#### Cellular states, lineage hierarchies, and transcriptional plasticity

Single-cell transcriptomic profiling has revealed that glioma organoids preserve the full spectrum of tumour cell states observed in vivo, including stem-like, progenitor-like, differentiated, and mesenchymal populations. Multidimensional atlases of glioblastoma organoids have identified coordinated transcriptional networks involving *SOX2*, *OLIG2*, *ASCL1*, and *NES* within neural stem–like compartments, alongside mesenchymal programmes characterised by *the expression of CHI3L1, CD44, STAT3, RELB, and YKL-40* [44]. These states coexist within individual organoids, recapitulating spatial and functional heterogeneity rather than collapsing into dominant phenotypes as seen in 2D cultures [45].

Cortical organoid–glioma co-culture systems have further demonstrated dynamic transcriptional plasticity, showing that glioblastoma cells undergo state transitions towards invasive and mesenchymal-like phenotypes when exposed to neural microenvironments, accompanied by upregulation of *VIM*, *FN1*, and *ZEB1* [46]. Single-cell lineage tracing and ribonucleic acid (RNA) velocity analyses revealed bidirectional interconversion between proneural and mesenchymal states, supporting the concept of reversible cell-state plasticity rather than rigid hierarchical differentiation [46, 47].

Emerging evidence further implicates non-coding RNA–mediated structural remodelling in driving transcriptional plasticity and resistance. Dynamic reorganisation of the long non-coding RNA *LINC01956* has been shown to enhance temozolomide resistance in *MGMT*-methylated glioblastoma organoids by modulating chromatin accessibility and the expression of deoxyribonucleic acid (DNA) repair genes, including *RAD51* and *BRCA1* [48]. These findings underscore the ability of organoids to capture higher-order regulatory mechanisms that are largely inaccessible in simpler models.

While these studies demonstrate the ability of organoids to capture diverse cellular states, most analyses rely on cross-sectional single-cell datasets rather than longitudinal sampling across treatment cycles. Consequently, although transcriptional plasticity is clearly observed, the long-term stability and reproducibility of these states across independent PDOs remain incompletely characterised [37]. Larger datasets incorporating temporal sampling and external validation will therefore be essential to confirm the generalisability of these findings.

#### Modelling TME interactions

Glioma progression is strongly influenced by the TME, including endothelial cells, immune infiltrates, ECM, and perivascular niches. Advanced organoid systems have enabled partial reconstruction of these interactions through co-culture and bioengineering strategies. Glioblastoma organoids have been shown to intrinsically preserve tumour-associated macrophage and microglia-like transcriptional programmes, including expression of *CSF1R*, *CX3CR1*, *AIF1*, and immunosuppressive cytokines, even in the absence of exogenous immune cells [49].

GLICO models have demonstrated that glioblastoma cells actively infiltrate neural tissue, migrate along neuronal tracts, and establish perivascular-like niches characterised by *VEGFA*, *ANGPT2*, and *PDGFB* signalling [47, 50].

Integration of immune cells and chimeric antigen receptor (CAR-T) therapies into patient-derived glioblastoma organoids has enabled real-time assessment of immune infiltration, cytotoxicity, and antigen escape, revealing heterogeneous responses driven by differential expression of *IL13RA2*, *EGFR*, and *B7-H3* [51, 52]. Organoid-based evaluation of next-generation CAR-T strategies demonstrates potent antitumour activity of *CD44*/*CD133* dual-targeting *IL7R*α-armoured CAR-T cells, which exhibit enhanced persistence, cytokine production, and cytotoxicity within heterogeneous glioblastoma organoids [53]. Similarly, rationally designed cocktail CAR-γδ T cell therapies targeting multiple antigens have shown robust tumour clearance across diverse glioblastoma organoid models, highlighting the value of organoids for overcoming antigen escape [54].

Beyond efficacy testing, brain organoids have been leveraged as tumourigenicity evaluation platforms for cell therapies, enabling assessment of off-target neural toxicity and aberrant proliferation prior to clinical translation [55]. These systems provide a powerful framework for interrogating tumours–immune dynamics that are poorly captured in xenograft models.

Nevertheless, current glioma organoid systems only partially recapitulate the complexity of the glioma TME observed in vivo. Glioblastoma is characterised by a highly specialised brain microenvironment involving abnormal vasculature, infiltrating immune cells, resident microglia, and complex metabolic interactions within the CNS [3, 8]. However, many patient-derived glioma organoid platforms lack functional vascular networks, blood brain barrier (BBB) components, and systemic immune circulation, limiting their ability to recapitulate key processes, such as tumour-associated angiogenesis, immune cell trafficking, and metabolic exchange, that occur within the brain tumour niche [5]. Although certain organoid models preserve tumour-associated microglial or macrophage transcriptional signatures, adaptive immune processes, including T-cell priming, antigen presentation dynamics, and systemic cytokine signalling, are often absent or only partially represented, which may reduce the ability of these models to fully capture immune-mediated tumour progression and treatment response [6]. These limitations are particularly relevant for glioblastoma, where immune suppression within the tumour microenvironment and interactions with infiltrating lymphocytes strongly influence therapeutic outcomes. Consequently, the absence of fully functional immune and vascular systems in many glioma organoid models may affect the accuracy of predicting responses to immunotherapies and anti-angiogenic treatments [15, 52]. Furthermore, although recent studies have incorporated immune components into glioblastoma organoid platforms to evaluate therapies such as CAR-T cells, these investigations remain largely limited to small experimental cohorts and preclinical validation, highlighting the need for larger translational studies that correlate organoid-based therapeutic responses with patient clinical outcomes [18, 52].

#### Applications in precision therapy; invasion, therapeutic resistance, vulnerabilities and drug screening

Glioma organoids have played a crucial role in elucidating the mechanisms underlying invasion and therapeutic resistance, serving as experimentally accessible models that maintain tumour architecture and cellular heterogeneity. Invasion assays employing brain organoid co-cultures reveal that glioblastoma cells migrate both collectively and individually within neural tissue, forming actin-rich protrusions and extensively remodelling the ECM through the activity of *MMP2*, *MMP9*, and *ITGB1* [46, 47]. Single-cell analyses following chemotherapeutic or radiotherapeutic interventions demonstrate an enrichment of mesenchymal-like, therapy-resistant phenotypes characterised by activation of *STAT3*, NF-κB, and DNA damage response pathways. These findings highlight the close association between invasive potential and therapeutic resistance as adaptive phenotypic responses within 3D tumour models [44, 56].

Beyond invasive behaviour, glioma organoids enable detailed mechanistic dissection of both intrinsic and acquired drug resistance under sustained therapeutic pressure. Functional drug testing within PDOs has demonstrated strong concordance between temozolomide response and clinical outcomes, validating organoids as predictive platforms for resistance modelling [37, 57]. Mechanistic insights from these systems have identified centromere protein U (*CENPU*) as a critical regulator of temozolomide resistance, whereby *CENPU* promotes ubiquitination-mediated degradation of *RPS3*, suppressing DNA damage–induced apoptotic signalling and facilitating tumour cell survival [58]. Additional resistance mechanisms uncovered using organoids include *HSD52*/*NONO*/*SFPQ*-mediated DNA repair that facilitates temozolomide tolerance [58], DCPS inhibition [59], and viral oncolysis using the Zika virus as a cytotoxic agent [60]. In parallel, inhibition of thioredoxin reductase 1 (*TrxR1*) using BS1801 has been shown to elevate reactive oxygen species, induce endoplasmic reticulum stress and alleviate treatment resistance in glioma organoids, highlighting redox homoeostasis as an actionable vulnerability [61].

Combination therapy strategies assessed in organoid systems further illustrate that resistance is dynamically modulated through pathway interactions rather than single-gene effects. Albumin-bound paclitaxel synergises with temozolomide by impairing homologous recombination repair through downregulation of *RAD51* and *BRCA2*, while simultaneously inducing ferroptosis via lipid peroxidation pathways, thereby overcoming chemoresistance in glioblastoma organoids [62]. Consistent with this, PDOs model treatment-induced DNA damage signalling, including sustained activation of *ATM*, *ATR* and *CHK1*, enabling longitudinal tracking of adaptive resistance trajectories under repeated therapeutic exposure [63].

High-resolution multi-omic profiling of glioblastoma-like organoids has further demonstrated that resistance is governed by coordinated oncogenic and metabolic networks rather than isolated pathways. Integrated transcriptomic, epigenomic and proteomic analyses reveal convergence on *MYC*-, *E2F*- and mTOR-driven programmes that sustain proliferation, stemness and metabolic flexibility during therapy [44]. Network-level analyses indicate that combinatorial targeting of cell-cycle regulators and metabolic dependencies yields superior cytotoxicity compared with monotherapies, reinforcing the value of organoids for systems-level optimisation of anti-resistance strategies [44].

Importantly, glioma organoids preserve tumour-intrinsic immune transcriptional programmes that contribute to therapy resistance independently of exogenous immune cells. PDOs retain interferon-responsive gene signatures, antigen presentation machinery (*HLA-A*, *HLA-B*, *B2M*) and cytokine signalling pathways enriched within progenitor-like and stem-like tumour populations and associated with non-genetic therapeutic tolerance [49]. These immune-intrinsic states persist under chemotherapeutic and targeted treatment pressure, modulating responses to DNA-damaging agents and contributing to heterogeneity in resistance. In parallel, super-enhancer-regulated drug-tolerant persister populations characterised by the *PPP1R15B*/*EIF2A* axis have been identified in glioblastoma organoids, revealing transcriptional mechanisms underpinning aggressive, therapy-resistant states and highlighting novel opportunities for targeting persistence under treatment [64].

Despite these advances, the clinical translation of organoid-guided precision therapy remains in its early stages. Many drug-screening studies involve limited patient numbers and retrospective analyses rather than prospective clinical trials [65]. Additionally, although predictive concordance with patient outcomes has been reported, the turnaround time for organoid generation and drug testing can still exceed the clinically actionable window for rapidly progressing tumours such as glioblastoma [37]. Standardisation of culture protocols, automation of screening workflows, and integration with genomic diagnostics will therefore be essential to enable real-time clinical implementation.

Organoids that preserve endothelial and TME interactions further extend resistance modelling beyond tumour-intrinsic mechanisms. Patient-derived systems capturing these interactions enable investigation of resistance to standard-of-care therapies, such as temozolomide-induced macrophage infiltration and microenvironment-mediated survival signals, which are difficult to recapitulate in conventional 2D cultures [66, 67]. Collectively, these findings underscore how glioma organoids integrate tumour-intrinsic molecular heterogeneity, immune programming and DNA repair dynamics to inform patient-specific precision oncology approaches [59, 60]

Glioma organoids have also enabled the identification of epigenetic dependencies within cancer stem cell compartments. *WDR5*, a core component of the MLL/SET1 histone H3K4 methyltransferase complex, sustains glioblastoma stem cell self-renewal, transcriptional plasticity and tumourigenicity through regulation of stemness-associated gene networks, including *SOX2*, *MYC* and *OCT4* [68]. Pharmacological inhibition or genetic depletion of *WDR5* selectively impairs stem-like populations within glioblastoma organoids, sensitises tumours to cytotoxic therapy and suppresses tumour propagation, establishing *WDR5* as a therapeutically exploitable epigenetic vulnerability effectively captured in organoid systems [68]. Similarly, LGG organoids have enabled subtype-specific drug testing, revealing selective vulnerabilities of *IDH*-mutant tumours to metabolic inhibitors and epigenetic modulators [39].

Emerging studies further demonstrate the utility of organoids for evaluating metabolic combination strategies. Inhibition of the hexosamine biosynthesis pathway using FR054 enhances temozolomide sensitivity by promoting ferroptosis and inhibiting O-GlcNAcylation, highlighting the potential of organoid-guided metabolic targeting approaches [69]. Organoid models have also facilitated functional testing of novel therapeutic strategies targeting resistance mechanisms, including monensin derivatives that disrupt ion homeostasis and doxorubicin-conjugated ultrasmall gold nanoparticles designed to enhance blood–brain barrier penetration [70, 71]. In parallel, targeting senescent neuron–tumour interactions has emerged as a promising strategy, with inhibition of prostaglandin E2 (PGE2) signalling enhancing therapeutic efficacy by disrupting tumour-supportive neuronal niches in glioblastoma organoid models [72].

As patient-derived models, glioma organoids enable functional evaluation of drug sensitivity and resistance within clinically relevant timeframes. Multiple studies demonstrate concordance between organoid drug responses and patient outcomes following temozolomide treatment, with predictive accuracies exceeding 80% in some cohorts [37, 57]. Organoid-based screening further identifies differential sensitivity to targeted inhibitors, including *EGFR* inhibitors, *CDK4/6* inhibitors and PI3K–AKT–mTOR pathway modulators, reflecting underlying molecular heterogeneity [5, 73]. Tumour organoid models of gliomas have also facilitated the evaluation of experimental biological approaches, such as oncolytic virotherapy, with the Zika virus demonstrating selective replication and oncolytic activity in aggressive glioma cells within these three-dimensional tumour organoid systems [60].

High-dimensional molecular profiling has further expanded the therapeutic landscape accessible through organoid models. A multidimensional atlas integrating transcriptomics, epigenomics, proteomics, and functional drug screening has revealed coordinated oncogenic networks governing proliferation, stemness, and metabolic adaptation, including convergence on MYC-, E2F-, and mTOR-driven regulatory programmes [44]. This atlas has enabled identification of context-specific drug sensitivities, uncovering compounds targeting *CDK7*, BET bromodomains and oxidative phosphorylation pathways, with responses varying across molecular subtypes and stem-like states preserved within organoids [44]. Notably, immune-related transcriptional states persist under therapeutic pressure and modulate responses to DNA-damaging agents and targeted inhibitors, thereby influencing resistance trajectories and treatment efficacy [49].

Advances in organoid engineering have further strengthened their translational relevance. High-throughput laboratory-engineered platforms enable scalable screening of hundreds of compounds while preserving transcriptional fidelity, facilitating identification of candidate therapeutics that may be overlooked in 2D assays [15, 42]. Hydrogel-based scaffolds such as GelMA–HAMA optimise glioblastoma organoid construction by supporting clonal growth, uniform architecture, and prolonged viability, thereby enhancing reproducibility in drug-screening applications [74]. PDOs established from stereotactic biopsy tissue enable rapid, clinically actionable drug testing even from limited or heterogeneous samples, facilitating personalised therapy selection and validation in preclinical settings [12, 41].

**Fig. 3:**
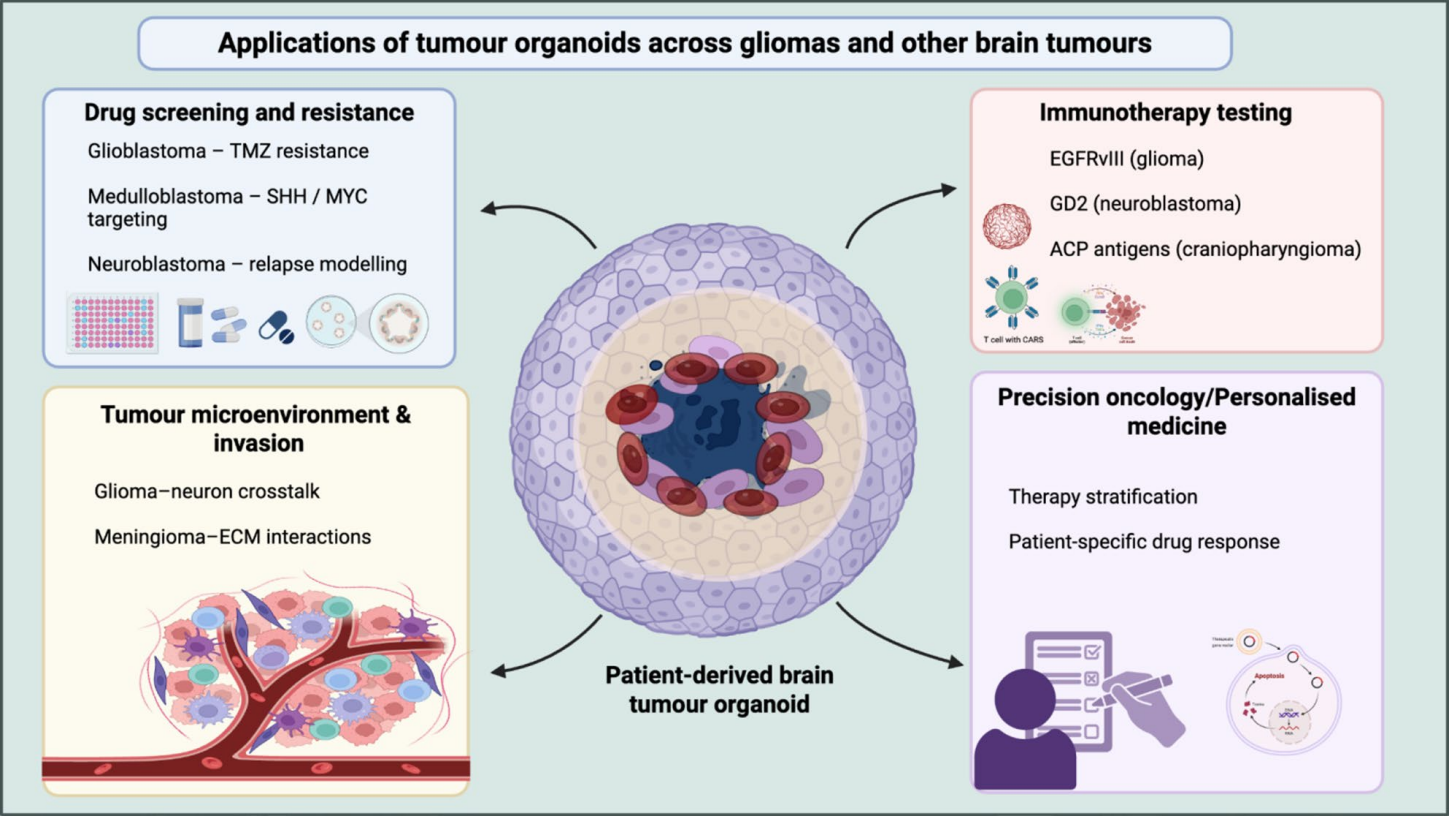
Applications of tumour organoids across gliomas and other brain tumour research (Image is created with Biorender.com). TMZ, Temozolomide; ACP, Adamantinomatous Craniopharyngioma; EGFR, Epidermal Growth Factor Receptor; GD2, Disialoganglioside 2; SHH, Sonic Hedgehog; MYC, MYC proto-oncogene; ECM, Extracellular Matrix

Finally, functional readouts and translational integration are further enhanced through methodological innovations. Flow cytometry-based protocols have been optimised for glioblastoma organoids to enable accurate assessment of cell death, apoptosis and cytotoxic responses to experimental therapies, supporting high-throughput mechanistic studies [75]. Organoid-based xenograft systems bridge in vitro and in vivo modelling, with human brain organoid xenografts demonstrating selective tumour cell ablation following photodynamic therapy while preserving surrounding neural tissue, underscoring the translational potential of organoid-guided therapeutic development [76]. Figure 3 summarises the applications of organoids across brain tumour research.

### Meningiomas

One of the major challenges associated with translating preclinical discoveries to clinical progress in the field of meningioma has been the inability of conventional experimental models to accurately mimic the biological complexity of the human tumour. In order to overcome this challenge, several studies have attempted to generate patient-derived meningioma organoids (MEN-Os) from tumour tissues resected during neurosurgical procedures. These models have been shown to retain several characteristics of the parental tumour tissues across different studies conducted by different investigators [12, 77–82]. In addition to these characteristics, immunohistochemical studies have demonstrated that these models retain several key characteristics of meningioma tumour tissues, including the progesterone receptor and somatostatin receptor-2 [80, 82]. Furthermore, some meningioma organoid systems have been shown to retain the TME, including immune cells like macrophages and T-cells, suggesting that these models may mimic the TME to some extent.

Despite such advances, the capacity of MEN-Os to recapitulate all aspects of the TME still remains limited. For example, the ability of the microenvironment to recapitulate the extracellular matrix, such as collagen-containing fibrous tissue, has been difficult to achieve, as demonstrated by the limited recapitulation of the microenvironment in some MEN-Os, as discussed by [81]. Moreover, the first reports of organoid-based research for the study of meningioma tumours were based on limited patient cohorts which may limit the reproducibility of the results for different types of meningioma [77, 78]. Recent advances in the microenvironmental conditions for the culture of MEN-Os, such as the use of xeno-free media, may address such concerns [81, 82].

MEN-Os have also been used to explore tumour biology and identify possible therapeutic vulnerabilities. Yamazaki et al., (2021) have demonstrated that the transcription factor forkhead box M1 (*FOXM1*) plays a central role in meningioma proliferation, with *FOXM1* overexpression promoting the growth of MEN-Os [78]. Thus, *FOXM1* overexpression was inhibited using *FOXM1* inhibitor thiostrepton. The aforementioned pharmacological intervention, when combined with radiotherapy, was shown to suppress proliferation in the organoid model [78]. The following studies have also supported the importance of this pathway. This has been corroborated by subsequent studies, which have reinforced the importance of this mechanism by demonstrating that the RAF inhibitor AZ628 inhibits tumour proliferation by targeting *FOXM1* [83]. Organoid cultures have also been used to identify tumour-initiating populations within meningiomas. Through the use of sc-RNA seq, a SULT1E1-positive subpopulation has been identified that is associated with aggressive behaviour of meningiomas, and functional studies in MEN-Os suggested that activation of *SIRT1* using the agonist SRT1720 may selectively target this subpopulation within meningiomas [79]. Additional studies have suggested molecular mechanisms underlying meningioma development, including *CDH2* and *PTPRZ1* as potential therapeutic targets [84].

More recently, organoid systems have also been employed for drug screening. In one of the largest studies conducted to date, Jungwirth et al., (2026) developed PDOs from 60 samples of meningioma and carried out a pharmacological screen with a set of anticancer compounds [85]. This screen identified the histone deacetylase inhibitor panobinostat as active in approximately 70% organoids along with mechanistic analyses implicating *HDAC1/2* inhibition and resistance mediated by *HDAC8* up-regulation. However, the clinical utility of these studies conducted using organoid systems remains unknown and needs to be validated prospectively against patient outcomes. Similar studies involving the use of MEN-Os to investigate new therapeutic strategies include the assessment of oncolytic viral-based treatments, including the use of Zika virus to eliminate tumour cells [60].

### Medulloblastoma

PDOs have been established as promising systems for modelling medulloblastoma biology because they preserve the major structural characteristics of parental tumours. Various studies have indicated that patient-derived medulloblastoma organoids retain tumour morphology and cellular composition, especially in aggressive group 3 medulloblastomas [86, 87]. In addition, transcriptomic studies of patient-derived 3D tumour models indicated that organoid models retain tumour characteristics that more closely resemble those of parental tumours than conventional 2D tumour cultures [88]. In some cases, the response to conventional chemo-radiotherapy has been reported to resemble outcomes observed in experimental settings using PDOs. However, these studies are based on limited experimental data and are insufficient to confirm the potential of PDOs as predictive tools for assessing patient outcomes.

Additionally, organoid technologies have helped to investigate new immunotherapeutic and virotherapeutic approaches in medulloblastomas. For instance, dual immune checkpoint blockade targeting *CD155/TIGIT* and *PD-1/PD-L1* pathways has been found to increase the cytotoxic activity of natural killer cells against medulloblastoma cells using an organoid-based approach [89]. Moreover, 3D models of tumours have helped to investigate oncolytic virotherapy using Zika virus to selectively replicate and induce tumour cell killing in highly aggressive CNS tumour cells cultured using an organoid approach [60]. Although these immunotherapy approaches have been successful in organoid models, existing models only partially mimic the immune microenvironment and are often constrained by the limited number of patient samples. Hence, their application is more to hypothesis generation than to prediction.

Organoids have not only provided valuable insights into how a patient responds to treatment but also helped define molecular drivers and therapeutic vulnerabilities in several types of medulloblastoma. Using human cerebellar organoid models, researchers have studied MYC-driven group 3 medulloblastomas and found that OTX2 and c-MYC are important oncogenic drivers of this cancer type; additionally, they have demonstrated that *SMARCA4* can inhibit tumour cell proliferation in these models [25]. This supports the idea that the upregulation of MYC is a mechanism that creates vulnerability to dual inhibition of CDK12 and CDK13 in high-risk group 3 medulloblastomas. Therefore, organoid systems can also be used to identify targeted therapeutic strategies for this aggressive cancer type [90]. Other studies have identified metabolic and epigenetic vulnerabilities in these tumours, such as dependence on NADH shuttles for mitochondrial function under hypoxic conditions [91], as well as growth inhibition with the dual *HDAC/PI3K* inhibitor CUDC-907 in MYC-dependent tumours [92]. Corroborating these findings, approaches to screen for small molecules have revealed EPLIN as another potential target for therapy in MYCN-amplified tumours, where organoids have shown to be sensitive to compounds targeting this pathway [93]

Additionally, organoid systems have helped in the investigation of specific therapeutic targets in Sonic Hedgehog medulloblastoma tumours (SHH-MB). In particular, explant organoid models derived from SHH tumours have helped investigate the OLIG2 inhibitor CT-179, based on evidence that OLIG2-positive stem-like cells contribute to tumour recurrence [94, 95].

However, some limitations exist in the application of the organoid system to medulloblastoma research. These include variability in the establishment efficiency of organoid models and an inability to fully recapitulate the TME, limitations commonly reported across various organoid systems [96]. Moreover, there is a lack of prospective studies demonstrating a correlation between in vitro drug response and patient outcomes [88]. In summary, although the organoid system is important for investigating tumour biology and identifying promising drugs for cancer treatment, its application in cancer precision medicine remains investigational, a limitation highlighted across different brain tumour organoid models [88, 93]. On the other hand, glioma organoids have already progressed towards becoming clinically relevant drug testing platforms, with patient-derived models demonstrating the ability to preserve tumour heterogeneity and transcriptional states [42, 43]. Furthermore, advances in genetic engineering have improved structural fidelity and functional readouts, thereby strengthening their translational relevance to precision oncology in relation to gliomas. Medulloblastoma organoid research has yet to attain this level of impact.

### Craniopharyngioma

Recent work suggests that organoid technology has the potential to help overcome a critical barrier to research on craniopharyngioma, particularly in adamantinomatous craniopharyngioma (ACP) research. This is primarily due to the complex morphology of these tumours and their associated signalling pathways, which cannot be modelled in a conventional 2D culture system. Recently, a biobank comprising 54 patient-derived ACP organoids and their associated patient samples was established for a large-scale ACP study [97]. It was shown that PDOs maintain the characteristic histoarchitecture, the localisation of β-catenin, and the majority of the somatic mutation profile of the patient's tumour. Hence, the establishment of PDOs enables the development of a model for studying ACP and provides expansion cultures [97]. However, as is often the case in this large platform study, only a few of the newly established lines had detailed molecular analyses. Additionally, drug screening was not feasible across all lines due to differences in growth rates. Zhang et al. reported the generation of expandable organoid cultures that retained key histopathological features, β-catenin signalling activity, and genetic fidelity [98]. They thus provided a useful in vitro platform for studying tumour heterogeneity and disease mechanisms in ACP and further demonstrated that organoid cultures preserved tumour-specific transcriptional programmes and cellular heterogeneity present in the parental tumour tissues [98].

In addition, previously reported craniopharyngioma organoids have shown value in preclinical therapeutic evaluation using a 3D organoid system, demonstrating that treatment responses differed substantially between 2D and organoid cultures. For example, while B7-H3-targeted CAR-T therapy demonstrated robust anti-tumour effects in 2D cultures, its reduced efficacy in 3D organoid models underscores the influence of cellular-level organisation on therapeutic response [99]. Thus, this study demonstrates differences in treatment outcomes between 2 and 3D systems. CAR-T cells appear to exhibit greater cytotoxicity in 2D "monolayer cultures" than in 3D "organoid cultures" [99]. The B7-H3 antibody–drug conjugate showed efficacy in both cultures. This difference demonstrates that 2D cultures may yield exaggerated estimates of efficacy due to their inability to model structural and organisational barriers and the restricted delivery of the therapeutic agent into the target tissue [99].

More recent transcriptomic analyses have further highlighted the potential of ACP organoids for therapeutic investigation. Using single-nucleus RNA sequencing and spatial transcriptomic profiling, Chen et al. [2024] identified significant overexpression of the receptor tyrosine kinase AXL in cancer-associated fibroblasts within ACP tumour samples [97]. Importantly, functional validation in patient-derived ACP organoids demonstrated that pharmacological inhibition of AXL reduced organoid growth and decreased PD-L1 expression, suggesting that targeting stromal–tumour signalling pathways may represent a potential strategy for combination immunotherapy approaches in ACP [97]. Additionally, studies characterising primary ACP cells with cancer-associated fibroblast features have emphasised the importance of tumour–stroma interactions in ACP biology and suggested that organoid-based co-culture systems incorporating fibroblast populations may provide improved platforms for modelling TME interactions and evaluating stromal-targeted therapies [97]. However, the translational conclusion remains limited due to the small cohort size.

Research advancements in this area continue to provide evidence supporting the potential to test drugs on ACP organoids and highlight that the field is still very early in development. The Chen et al. [97] study on Axl as a therapeutic target highlights that these models may be used primarily to screen for new drugs and cannot transition immediately into clinical practice. Furthermore, several studies have highlighted that functional precision oncology in the care of patients with CNS tumours is limited by delays in turning around results of experiments, inadequate models of immune and vasculature microenvironments, differences in standardisation between labs, and limited data available to validate treatment outcomes from prospective studies [97, 100]. These findings highlight the importance of tumour architecture in shaping therapeutic response and support the evidence base for using organoid models as clinically relevant platforms for drug testing and precision medicine approaches in craniopharyngiomas.

However, on comparing craniopharyngioma with other intracranial tumours, especially gliomas and medulloblastomas, the application of organoids systems research remains relatively limited. In medulloblastoma, organoid technology has been extensively utilised to study subtype-specific drivers of tumorigenesis and therapeutic opportunities, including MYC-driven group 3 medulloblastomas and the role of the Sonic Hedgehog signalling pathway in a subgroup of medulloblastomas, which has led to the identification of metabolic vulnerabilities and candidate therapies [25, 90, 91, 93]. Likewise, glioma organoid cultures have been more advanced in their translational potential, where research has been conducted on the use of multi-dimensional molecular profiling and high-throughput drug screening approaches to understand the specific drug sensitivities and oncogenic signaling in each case [44]. These differences highlight the need for further methodological development and clinical validation before organoid platforms can be widely applied to precision medicine approaches in craniopharyngioma.

### Pituitary tumours

3D organoid models have become widely used tools for investigating pituitary tumours, now classified as pituitary neuroendocrine tumours (PitNETs). These patient-derived PitNET organoids faithfully reproduce key characteristics of the original lesions, including diverse nuclear morphologies and the expression of pituitary stem cell markers such as *SOX2*, *S100β*, *KRT8/18*, as well as other tumour-associated genes [101]. Pituitary organoid systems further emphasise that these models recapitulate aspects of pituitary gland architecture and stem cell niches, enabling investigation of pituitary development, tumourigenesis, and endocrine signalling pathways in both healthy and diseased tissues, thereby establishing organoids as versatile platforms for studying pituitary stem cell biology and tumour initiation [101]. Advances in culture techniques have significantly increased the success rate of organoid establishment to approximately 87% and have enabled these models to preserve subtype-specific hormone expression, characteristic mutations, and the microenvironmental heterogeneity intrinsic to the original PitNETs [102]. In this study, PDOs from multiple PitNET subtypes were successfully expanded and used for pilot drug screening, demonstrating that therapeutic responses can vary substantially between individual patients and highlighting the potential of organoids for personalised therapeutic testing. However, the authors also note that these findings were derived from a relatively limited patient cohort, indicating that larger multi-centre studies will be necessary to validate reproducibility and clinical applicability [102]. Molecular studies also suggest that aggressive behaviour in specific functional subtypes may be influenced by epitranscriptomic mechanisms; for example, *FTO*-mediated m6A demethylation of *DSP* facilitates the development of an aggressive subtype of growth hormone–secreting PitNETs [103]. These molecular insights highlight the ability of organoid-compatible genomic and epitranscriptomic analyses to uncover tumour subtype–specific regulatory pathways, although functional validation in organoid models remains limited and requires further investigation in larger experimental cohorts [103].

Consequently, pituitary tumour organoids are increasingly utilised in translational research, facilitating patient-specific disease modelling and pharmacological testing. This is particularly relevant for clinically challenging subtypes such as dopamine agonist–resistant prolactin PitNETs, where resistance mechanisms and alternative therapeutic strategies are actively being investigated [104]. Drug resistance in prolactin PitNETs emphasises that 3D tumour models, including organoids, may provide improved platforms for studying mechanisms of dopamine agonist resistance and identifying novel therapeutic targets, particularly in cases where conventional cell culture systems fail to reproduce clinically observed resistance phenotypes [104]. For example, organoids derived from adrenocorticotropic hormone (ACTH)-secreting pituitary tumours associated with Cushing’s disease have demonstrated the ability to secrete ACTH in vitro and to retain the genomic alterations observed in the patient’s tumour [105]. These organoid systems were also shown to maintain functional endocrine activity over extended culture periods, supporting their use as physiologically relevant models for investigating hormone secretion dynamics and therapeutic response in corticotroph tumours [105]. Consistent with their role as functional platforms for drug testing, patient-derived PitNET organoids have also been used to assess targeted therapies, with ceritinib shown to inhibit organoid growth and reduce ACTH production [106]. Importantly, this study highlights the translational potential of PDOs for testing targeted kinase inhibitors in endocrine tumours; however, the experimental validation was performed on a limited number of organoid lines, underscoring the need for larger organoid biobanks to improve statistical robustness and reproducibility of drug-response findings [106].

Researchers have developed pituitary tumour organoids derived from PSCs to investigate tumour biology. By directing hiPSCs through pituitary lineage differentiation and introducing key driver mutations such as *USP8* or *USP48* (commonly found in Cushing’s disease corticotroph tumours) scientists have created organoids that replicate corticotroph PitNETs. These engineered organoids exhibit features characteristic of pituitary tumours, including elevated ACTH and *TBX19* expression, suppression of other pituitary lineages, a high proliferative index, and expression of neuroendocrine tumour markers such as synaptophysin [107]. These genetically engineered organoid systems provide a powerful platform for mechanistic studies by enabling controlled manipulation of tumour driver mutations and endocrine lineage specification; however, they may not fully capture the complex mutational heterogeneity and TME interactions observed in spontaneous human PitNETs [107]. Notably, drug studies using these models have mirrored the differential responses seen in patients: for instance, PitNET organoids with *USP8*/*USP48* mutations respond to the selective glucocorticoid receptor modulator relacorilant with tumour cell apoptosis and minimal hormone release, whereas mifepristone, an antagonist, triggers increased ACTH secretion and cell proliferation [107]. These results highlight the potential of organoid models to accurately reflect pituitary tumour behaviour and inform personalised treatment approaches.

Foundational work in pituitary organoid research further supports the role of stem cell–derived organoid systems in modelling tumourigenesis. Early studies using murine tumourigenic pituitary models demonstrated that organoids derived from Drd2 pituitary tissue recapitulated stem cell activation and proliferative responses associated with tumour development, providing a framework for later human tumour organoid studies [108]. Subsequent research extended these findings to human pituitary tumours, in which tumour-derived organoids were shown to exhibit stemness-associated phenotypes and tumour-specific transcriptional programmes, thereby enabling investigation of the role of pituitary stem cells in tumour initiation and progression [109]. However, despite these advances, most pituitary organoid studies remain limited by relatively small sample sizes and methodological variability across laboratories, which may affect the reproducibility of organoid establishment and long-term culture stability. Consequently, while PitNET organoids demonstrate strong potential for translational research and personalised medicine, further standardisation of culture protocols and the development of large multi-institutional organoid biobanks will be essential to ensure reproducibility and clinical readiness of these platforms [101, 102, 106]. Table 1 summarises brain tumour organoid models with their various platforms, biological features, applications, and limitations.

**Table 1:** Brain Tumour Organoid Models: Platforms, Biological Features, Applications, and Limitations: This overview summarises the use of 3D modelling platforms in the study of major brain tumours

Tumour type	Model platforms (rationale and distinctions)	Source material	Retained biological features	Main applications	Key limitations
Glioblastoma	*PDOs*: self-organising 3D cultures derived from patient tumours that preserve intratumour heterogeneity, cancer stem-cell populations, and molecular profiles. *Glioblastoma tumouroids*: engineered tumour constructs often grown in scaffolds or matrices that allow controlled modelling of tumour architecture, ECM interactions, and invasion dynamics. *Cerebral organoid–glioma co-culture models (GLICO/assembloids)*: tumour cells integrated with brain organoids to replicate tumour infiltration into neural tissue and neuron–tumour interactions. *Genetically engineered cerebral organoids*: CRISPR-modified neural progenitors generating tumours within developing brain organoids to study early oncogenesis and driver mutations. *Orthotopic PDOX models*: transplantation of patient-derived organoids into mouse brains to recreate in vivo tumour growth and vascular interactions. *Hydrogel scaffold and 3D bioprinted models*: engineered systems replicating mechanical properties of the brain ECM and spatial tumour organisation. *Microfluidic tumour-on-chip systems*: microengineered platforms allowing perfusion and controlled microenvironment gradients to study drug delivery and tumour–vascular interactions	Fresh surgical resections; stereotactic biopsy tissue; patient-derived tumour stem cells; iPSCs genetically engineered with glioblastoma drivers	Intratumour heterogeneity; cancer stem-cell hierarchy; TME interactions; invasive growth into brain-like tissue; transcriptional and genomic tumour profiles; therapy-resistant clones; partial immune signalling; tumour-vascular interactions	Precision oncology drug screening; testing chemotherapy and radiotherapy responses; modelling tumour invasion and migration; investigation of treatment resistance (e.g., temozolomide resistance); evaluation of immunotherapies (CAR-T, immune checkpoint strategies); discovery of new molecular targets	Limited immune system representation; incomplete vascularisation and perfusion; culture variability; partial loss of stromal components; time-intensive generation; high cost; inability to fully model systemic tumour–host interactions
Diffuse glioma (mixed grades)	*PDOs*: maintain tumour heterogeneity and glioma stem-cell populations for studying tumour progression. *Tumour spheroids*: simpler multicellular aggregates that enable faster and scalable 3D culture for drug testing. *3D co-culture systems*: allow investigation of tumour–stromal or tumour–glial interactions involved in tumour progression. *Microfluidic tumour models*: provide controlled environments to study tumour invasion, nutrient gradients, and therapy diffusion. *Orthotopic xenografts*: transplantation into animal brains enables in vivo investigation of tumour growth and therapeutic response	Surgical tumour tissue; glioma stem cells; biopsy samples	Glioma stem-cell populations; tumour heterogeneity; invasive growth patterns; partial tumour–stromal interactions; genomic alterations characteristic of diffuse gliomas	Investigation of glioma biology; evaluation of targeted therapies; study of tumour progression and cellular plasticity; drug screening	Loss of some microenvironmental components; incomplete immune modelling; variability in culture conditions; limited long-term stability
LGG	*PDOs*: preserve IDH-mutant tumour genetics and early tumour architecture relevant to slow-growing gliomas. *3D tumour cultures*: scalable systems enabling drug testing and molecular studies when organoid growth is limited. *Orthotopic xenografts*: provide in vivo modelling of tumour growth and therapy response where in vitro models grow slowly	Surgical resection samples; biopsy-derived tumour cells	Retention of key molecular alterations (e.g., IDH mutations); tumour cell populations; early tumour architecture	Study of tumour initiation and early progression; evaluation of targeted therapies; personalised drug testing	Slow growth limiting model scalability; difficulty maintaining long-term cultures; incomplete microenvironmental representation
Medulloblastoma	*Cerebellar organoid tumour models*: replicate cerebellar developmental context from which medulloblastoma originates. *Genetically engineered organoids*: allow introduction of subgroup-specific oncogenic drivers to study tumour initiation. *Paediatric tumour organoid platforms*: maintain tumour heterogeneity for drug testing. *Orthotopic xenografts*: allow in vivo evaluation of tumour growth and therapeutic response	Stem cells engineered with medulloblastoma driver mutations; patient tumour tissue	Developmental lineage features; subgroup-specific transcriptional programmes; tumour heterogeneity	Study of tumour initiation from cerebellar progenitors; investigation of developmental oncogenesis; testing subgroup-specific therapies	Difficulty modelling full TME; limited immune interactions; variability in differentiation states
Meningioma	*PDOs*: maintain tumour architecture and transcriptional signatures of meningioma tissue. *Hydrogel-based 3D cultures*: scaffold-supported systems that enhance tumour–matrix interactions. *TME-preserving organoids*: designed to retain stromal and immune components from the original tumour tissue	Surgical tumour tissue; primary tumour cells	Tumour cellular architecture; aggressive cell subpopulations; transcriptional signatures; tumour–microenvironment interactions	Investigation of meningioma pathophysiology; molecular target discovery; testing targeted therapies	Limited immune representation; difficulty modelling long-term tumour evolution; inter-patient variability
Pituitary tumours	*Pituitary organoid cultures*: replicate hormone-secreting endocrine cell populations found in pituitary adenomas. *Stem-cell-derived pituitary models*: generated from pluripotent stem cells to study tumour initiation and endocrine differentiation pathways. *Xenograft systems*: allow investigation of tumour growth and endocrine activity within a living organism	Surgical pituitary adenoma tissue; pituitary progenitor cells	Hormone-secreting cell populations; tumour cell heterogeneity; endocrine signalling pathways	Study of pituitary tumour biology; endocrine regulation; evaluation of targeted therapies	Limited studies; difficulty maintaining hormone-secreting phenotypes long-term; lack of full hypothalamic–pituitary axis context
Craniopharyngioma	*Organoid models*: reproduce tumour epithelial cell populations and tissue organisation. *Stem-cell-engineered tumour models*: enable study of developmental signalling pathways and driver mutations (e.g., WNT/β-catenin). *Xenografts*: allow evaluation of tumour growth and therapy responses in vivo	Patient tumour tissue; genetically engineered stem cells	Tumour epithelial cell populations; developmental signalling pathways (e.g., WNT/β-catenin)	Study of tumour pathogenesis; identification of molecular drivers; evaluation of targeted therapies	Limited model availability; difficulty recapitulating cystic tumour architecture; rarity of tumour limiting sample availability

It covers tumour types, model systems, source materials, retained biological features, principal research applications and key limitations. It also highlights differences in cellular origin, architectural complexity and degree of tumour microenvironment representation across platforms, including patient-derived organoids, tumour spheroids, engineered cerebral organoids, xenograft models, hydrogel/bioprinted systems and microfluidic tumour-on-chip technologies

PDO, Patient-Derived Organoid; PDOX, Patient-Derived Orthotopic Xenograft; GLICO, Glioma–Cerebral Organoid Co-culture; iPSC, Induced Pluripotent Stem Cell; LGG, Low-grade Glioma; CAR-T, Chimeric Antigen Receptor T Cells; TME, Tumour Microenvironment; IDH, Isocitrate Dehydrogenase; 3D, 3-Dimensional

## Advantages of tumour organoids over 2D cell cultures, xenograft models and genetically engineered mouse models for brain tumours

### Superior recapitulation of tumour heterogeneity and microenvironment

2D cultures, commonly derived from immortalised cell lines, fail to reproduce the 3D cellular architecture, cell–cell interactions, and gradients of oxygen, nutrients, and metabolites present in vivo [110, 111]. Tumour organoids, in contrast, maintain the hierarchical organisation of cancer stem-like cells, differentiated progeny, and niche-supporting cells, preserving both inter- and intratumoural heterogeneity [5, 7]. For glioblastoma, organoids retain transcriptional programmes characteristic of classical, mesenchymal, and proneural subtypes over multiple passages, a feature that 2D cultures and conventional xenografts rarely recapitulate [4, 43]. PDOs also preserve tumour-specific ECM composition, enabling realistic modelling of invasion, migration, and therapy resistance [11, 112].

### Improved modelling of tumour–microenvironment interactions

Xenograft models, including PDXs and zebrafish orthotopic models, partially maintain tumour architecture but are limited by interspecies differences, particularly in immune components and stromal interactions [113–115]. GEMMs allow the study of tumour initiation and progression within a genetically controlled context but cannot fully replicate the heterogeneous genetic landscape of human tumours [116, 117]. Tumour organoids overcome these limitations by incorporating human-specific cellular and microenvironmental features. For instance, organoid co-culture systems allow the integration of patient-derived immune cells, endothelial cells, and astrocytes, enabling the study of immune evasion, angiogenesis, and tumour–stroma crosstalk in a controlled in vitro setting [12, 13].

### Personalised medicine and predictive therapeutic response

A critical limitation of 2D cell lines and GEMMs is their poor translational predictability. Drug responses in 2D cultures often fail to correlate with patient outcomes due to a lack of 3D architecture, heterogeneity, and microenvironmental cues [7, 9]. Similarly, xenografts may require months to establish, and immune-deficient hosts limit evaluation of immunotherapies [111, 118]. Tumour organoids, by contrast, can be derived from patient tissue within weeks and preserve the molecular and cellular diversity necessary for personalised treatment testing [4, 15]. In glioblastoma, PDOs have demonstrated predictive accuracy exceeding 80% for temozolomide response, enabling real-time tailoring of therapeutic regimens [4]. Organoid models have similarly been used for paediatric medulloblastomas, diffuse intrinsic pontine glioma (DIPG), and LGGs, preserving subtype-specific features critical for individualised therapy development [5, 6, 12]

### High-throughput screening and multi-omics integration

GEMMs and xenografts are inherently limited in scalability for large-scale drug screens or multi-omics studies due to cost, ethical constraints, and temporal demands [119, 120]. In contrast, organoids are amenable to high-throughput drug screening, CRISPR-based functional genomics, and spatial transcriptomics, while maintaining clinically relevant tumour architecture [6, 14, 15]. This enables systematic interrogation of drug combinations, resistance mechanisms, and novel therapeutic targets across diverse brain tumour types, including glioblastoma, anaplastic astrocytomas, and oligodendrogliomas [28, 113].

### Modelling tumour evolution and resistance mechanisms

Longitudinal studies of tumour evolution and therapy resistance are challenging in 2D cultures and GEMMs. Organoids retain clonal diversity, allowing the study of subclone selection, mesenchymal transition, and therapy-induced resistance [4, 43]. For example, glioma organoids cultured under temozolomide or radiation treatment exhibit expansion of therapy-resistant clones, recapitulating patient relapse patterns more accurately than 2D or xenograft models [5, 12].

### Versatility across brain tumour types

Beyond glioblastoma, organoids have demonstrated utility in modelling paediatric brain tumours, including medulloblastoma, atypical teratoid/rhabdoid tumour, and ependymomas, as well as metastatic brain lesions, maintaining patient-specific heterogeneity and response profiles [3, 5, 6]. This versatility surpasses 2D cultures, which are often limited to established cell lines, and GEMMs, which require extensive genetic engineering for each tumour type [11, 14].

**Fig. 4:**
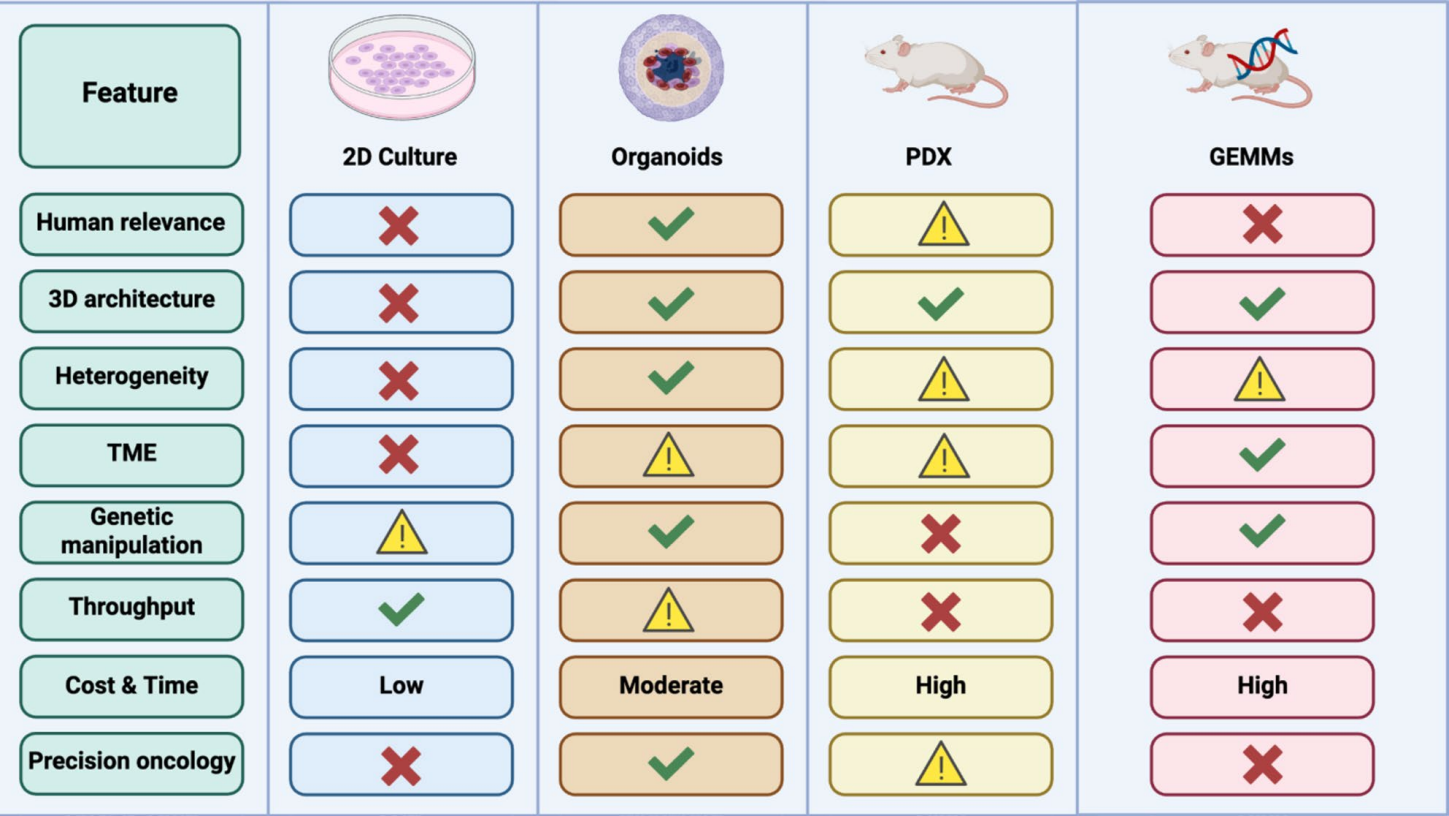
Comparative landscape of preclinical brain tumour models. Relative strengths and limitations of 2D cultures, organoids, PDX, and GEMMs across features including human relevance, 3D architecture, and tumour microenvironment (Image is created with Biorender.com). Abbreviations: 2D, Two-Dimensional; GEMM, Genetically Engineered Mouse Model; 3D, Three-Dimensional; PDX, Patient-Derived Xenograft; TME, Tumour Microenvironment. Symbols: ✔ strong suitability; ⚠ partial or context-dependent suitability; ✖ limited or absent suitability

In summary, tumour organoids provide a highly informative preclinical model for brain tumours by accurately preserving heterogeneity, modelling TME interactions, enabling personalised therapy prediction, supporting high-throughput and multi-omics analyses, and allowing longitudinal studies of tumour evolution and resistance. These advantages position organoids as a transformative tool in neuro-oncology research, bridging the translational gap inherent in 2D cultures, xenograft models, and GEMMs [3, 4, 6]. Figure 4 summarises the comparative analysis of tumour organoids over 2D cell cultures, xenograft models and genetically engineered mouse models for brain tumours.

## Discussions and prospects

### Developing methods to incorporate immune cells and form vascular networks

A significant limitation of classical organoid systems is the absence of functional vasculature and immune components, which are essential for modelling drug delivery, hypoxia gradients, and tumour–immune interactions. Recent work has demonstrated that incorporation of endothelial progenitor cells or mesodermal progenitors generates functional vascular networks within cerebral organoids, improving nutrient diffusion, reducing central necrosis, and sustaining proliferative niches analogous to in vivo tumours [121–125]. Vascularised organoids facilitate studies on BBB permeability and angiogenesis, critical for understanding glioma progression and therapeutic resistance [126, 127].

The integration of immune cells, including microglia, T cells, and peripheral monocytes, enables modelling of tumour–immune crosstalk and evaluation of immunotherapeutic interventions. Patient-derived tumour–immune organoids accurately recapitulate the immunosuppressive microenvironment of glioblastoma and predict responses to CAR-T therapy and immune checkpoint inhibitors, with correlations to patient outcomes exceeding 80% in some studies [4, 128, 129]. Such systems allow functional testing of patient-specific immune responses, addressing a key limitation of xenograft and 2D culture models, which lack the full spectrum of human immune interactions [3, 126].

### Creating more complex “brain-in-a-dish” models

To more faithfully recapitulate the glioma microenvironment, researchers have developed individualised patient tumour organoids (IPTOs), in which patient-derived tumour fragments are embedded within regionally specified cerebral organoids. These models maintain tumour heterogeneity, including stem-like and differentiated populations, and preserve spatial and functional interactions with surrounding neural tissue [130, 131]. IPTOs exhibit proliferative indices (Ki-67 labelling 25–40%) and invasion patterns consistent with patient tumours, enabling precise assessment of therapeutic efficacy and tumour progression [3, 4]. By recapitulating native niche architecture, IPTOs provide unparalleled insights into glioma invasion, heterotypic cell interactions, and microenvironmental adaptation, which are not achievable in conventional 2D cultures or xenograft models [127].

### Integration with other technologies: CRISPR editing, single-cell and spatial OMICs, AI

The integration of organoid platforms with CRISPR-Cas9 genome editing allows precise manipulation of oncogenes, tumour suppressors, and epigenetic regulators in patient-derived models, enabling the study of tumourigenesis, drug resistance, and synthetic lethality [132–134]. CRISPR-engineered organoids facilitate high-resolution functional screens to identify driver mutations and therapeutic vulnerabilities in glioblastoma stem cells, complementing single-cell and spatial transcriptomic analyses [3, 135].

Combining organoids with sc-RNA seq and spatial OMICs provides a comprehensive map of intratumoural heterogeneity, cellular states, and microenvironmental niches. Such approaches have revealed transcriptionally distinct subpopulations, spatially restricted immune microenvironments, and therapy-resistant clones, which can be modelled and tracked longitudinally in vitro [3, 4, 135]. Integration with AI-based analytics further allows automated morphological and functional evaluation, improving the accuracy of predicted drug responses and enabling high-throughput screening [136–139].

### Improving culture methods

Advances in organoid culture techniques, such as the air–liquid interface (ALI) and microfluidic systems, enhance growth, longevity, and structural fidelity. ALI cultures improve oxygenation and support long-term maintenance of neuronal and tumour diversity, maintaining functional synapses and electrophysiological activity [3, 140]. Microfluidic platforms enable controlled nutrient perfusion, gradient formation, and high-content imaging, facilitating quantitative assessment of proliferation, invasion, and therapeutic response [141, 142]. Optimised media formulations, including BrainPhys and neurotrophin-enriched media, support proliferation and differentiation, preserving tumour-like architecture and functional heterogeneity [143, 144]. These refinements increase organoid survival by 30–50% and enhance the translational relevance of experimental results.

### Biobanking

The establishment of large-scale organoid biobanks represents a critical step for reproducible research and precision medicine. Biobanks containing diverse brain tumour types, including glioblastoma, medulloblastoma, and diffuse midline gliomas, preserve interpatient heterogeneity and rare subclonal populations [37, 145, 146]. Cryopreservation techniques maintain post-thaw viability above 80%, preserving molecular, phenotypic, and functional characteristics [3, 130]. Such repositories enable large-scale drug screening, multi-institutional collaborative studies, and development of personalised therapeutic strategies. Integration of biobank data with genomic, transcriptomic, and phenotypic analyses facilitates patient stratification and accelerates the development of predictive models of therapy response [3, 4, 135].

Future advances in brain tumour organoid research will likely require on enhancing physiological fidelity through vascularisation, immune incorporation, and IPTOs; integration with multi-omic and AI technologies; improvements in culture methods via ALI and microfluidics; and the creation of comprehensive biobanks. These developments promise to transform preclinical modelling, improve predictive accuracy of patient-specific therapies, and ultimately bridge the translational gap from bench to bedside in malignant brain tumours [3, 4, 135].

## Study limitations

Although a comprehensive search strategy was employed, the restriction to English-language publications may have excluded relevant studies. In addition, the rapidly evolving nature of tumour organoid technologies means that emerging evidence, particularly from preprints and recently developed platforms, may not have been fully captured. The substantial heterogeneity across included studies, including differences in organoid derivation methods, culture conditions, tumour types, and analytical approaches, limits direct cross-study comparability. Furthermore, the technical complexity, high cost, and variable success rates of organoid generation restrict the generalisability of findings to specialised, well-resourced laboratories, potentially introducing selection bias. Finally, incomplete representation of key microenvironmental components, such as vasculature and immune cells, and variability in spatial and temporal resolution may influence the interpretation of tumour heterogeneity, invasion, and therapeutic response.

## Conclusion

Collectively, these studies indicate that brain tumour organoids are a reliable and translationally valuable platform for studying therapeutic resistance, actionable vulnerabilities, and heterogeneity in drug response within brain tumours. By preserving tumour-intrinsic molecular programs, stem cell hierarchies, immune-related transcriptional states, and microenvironmental interactions, organoids enable the mechanistic dissection of both intrinsic and acquired resistance to standard and experimental therapies. At the same time, organoid systems reveal context-specific genetic, epigenetic, metabolic, and redox vulnerabilities that can be therapeutically exploited, including dependencies within cancer stem cell compartments and adaptive stress response pathways. Importantly, integrating high-throughput drug screening, multi-omic profiling, and functional validation within patient-derived organoids provides a promising framework to inform precision oncology, supporting the exploration of clinical drug response, optimisation of combination strategies, and rational prioritisation of personalised therapies. Together, these advances position brain tumour organoids as an emerging link between molecular discovery and patient-specific treatment selection, with potential implications for addressing therapeutic resistance and informing clinical decision-making in brain tumours.

## Data Availability

No datasets were generated or analysed during the current study.

## Abbreviations

BTO: Brain tumour organoid
GSC: Glioma stem cell
CNS: Central nervous system
ECM: Extracellular matrix
PDO: Patient derived organoid
2D: 2-Dimensional
GEMM: Genetically engineered mouse model
3D: 3-Dimensional
PSC: Pluripotent stem cell
GFP: Green fluorescent protein
RNA: Ribonucleic acid
DNA: Deoxyribonucleic acid
GLICO: Cerebral organoid glioma
CAR‑T: Chimeric antigen receptor T
TME: Tumour microenvironment
CENPU: Centromere protein U
LGG: Lower-grade glioma
FOXM1: Forkhead box M1
SHH-MB: Sonic Hedgehog medulloblastoma
MEN-O: Meningioma organoid
PDX: Patient-derived xenograft
KSP: Kinesin spindle protein
ACP: Adamantinomatous craniopharyngioma
PitNET: Pituitary neuroendocrine tumour
ACTH: Adrenocorticotropic hormone
hiPSC: Human induced pluripotent stem cell
IPTO: Individualised patient tumour organoid
ALI: Air–liquid interface
